# “The very interesting finding suggests that…”: A cognitive frame-based analysis of interest markers by authors’ geo-academic location in applied linguistics research articles

**DOI:** 10.3389/fpsyg.2022.1020854

**Published:** 2022-10-26

**Authors:** Qian Wang

**Affiliations:** School of Foreign Studies, Northwestern Polytechnical University, Xi'an, China

**Keywords:** linguistic expressions of interest, interest markers, frame semantics, geo-academic location, research article

## Abstract

Linguistic expressions of interest instantiated by *interesting*, *intriguing*, and *fascinating* that signal the authorial stance are not uncommon in applied linguistics research articles. Nevertheless, they have received little scholarly attention. This paper, taking a cognitive semantic approach, reports on a study that sought to examine how linguistically expressed interest in applied linguistics research articles is leveraged by researchers’ geo-academic location (the Core vs. the Periphery). Drawing on a semantic frame generated for interest markers in academic writing, this study focused on the incidence of the various elements of the Interest frame in the discipline of applied linguistics based on a mixed-methods approach. The corpus-based quantitative analyses found that academic writers’ geo-academic location was a robust predictor of authors’ overall use of interest markers and some frame elements associated with the Interest frame. Triangulation with the interview data obtained from disciplinary specialists revealed that the observed differences could be attributable to the hierarchical academia featuring periphery-based scholars’ unequal access to the knowledge production market and under-representation.

## Introduction

Academic writing generates and disseminates disciplinary knowledge in a specific field of study. Research articles (RAs), as a prestigious academic genre and rhetorically sophisticated artifacts for generating new knowledge and communicating scientific inquiries (Hu and Cao, 2015), are central to academic work and a robust indicator of the achievements of a certain academic standard. Traditionally, RAs are expected to be objective, faceless, and impersonal and seen as an embodiment of detached reasoning and rationality. However, it has now been regarded as a “privileged form of argument” (Hyland, 2011, p. 193) deployed by academic writers to encode concepts, express authorial attitudes, and interact with or engage the intended audiences to persuade them (Hyland, 2020). As such, attitude markers are essential linguistic resources to achieve writer-reader interaction (Hyland, 2005) because they allow “writers to both take a stand and align themselves with disciplinary-oriented value positions” (Hyland, 2011, p. 199). In applied linguistics RAs, it is quite common to encounter expressions of interest (hereafter interest markers) instantiated by, for example, *interesting*, *intriguing*, and *fascinating*. This emotive response is characterized as a knowledge emotion (Silvia, 2010) because it motivates our epistemic efforts to resolve cognitive incongruence stemming from perceived discrepancies between new information received and prior knowledge (Silvia, 2019). Thus, interest markers, as a sub-type of attitude markers (Hyland, 2005; Hu and Chen, 2019; Chen and Hu, 2020), are inherently associated with knowledge-making practices in academic writing.

Academic writing, although involving a huge number of scholars, publishers, and research/higher education institutions across the globe, are locally situated practices and context-bound (Howells, 2012; Hyland, 2015). Consequently, academic writing is possibly influenced by, for example, the sociocultural environment where scientific communication occurs or even academics’ research institutions that are geographically defined. Researchers in China, for example, share certain experiences of academic training and international publications with researchers elsewhere. However, their attitudes, epistemological beliefs, and academic practices might also be shaped by the norms valued in the local community where they conduct their research or the research conventions of their institutional affiliations. Due to the imbalanced distribution of global academic resources, the Core^1^ (e.g., North America/European countries) dominates the production and dissemination of academic knowledge (Kieńć, 2017; Larson, 2018). Notably, when institutional policies give more credit to Center-affiliated journals in which English has become unquestionably the language for scientific exchange, this linguistic imperialism (Canagarajah, 2002; Pennycook, 2017; Lillis and Curry, 2018) could influence regional intellectual and epistemological assumptions espoused by scholars in general and discursive practices such as expressing attitudes in particular. Some research has found that scholarly practices acquired in geographically defined research sites could be manifested in the presentation and organization of knowledge with community-preferred patterns of alignment (Swales, 1996; Yakhontova, 2002; Tight, 2007).

However, to date, there is very limited research on the potential influences of geo-academic locations on academics’ discursive practices, particularly on such impacts brought about by a specific type of authorial attitude, for example, expressing interest in scientific discourse. Given that the epistemic nature of interest markers is inherently connected with knowledge-making in RAs, this study, taking a cognitive semantic approach, set out to examine how the use of interest markers in applied linguistics RAs may be mediated by researchers’ geo-academic locations. This attempt is expected to unveil the possible knowledge-making practices that underpin academic traditions and values in the field of applied linguistics within the global context. Two research questions were proposed to guide the study.

1. Are there any differences in the overall use of interest markers between applied linguistics RAs written by academic writers affiliated with geographically defined research communities?
2. Are there any differences in the use of interest markers between applied linguistics RAs written by academic writers affiliated with geographically defined research communities in terms of the distribution of each frame element category?

## Previous research

### Interest markers as evaluative resources in academic writing

Linguistic expressions of interest in writing index the writer’s stance toward advanced propositions and, more importantly, the writer’s social presence in discourse. They are part of emotive communication, purporting to interact, engage, or persuade readers strategically. To date, most studies revolving around evaluative resources have been conducted primarily from a metadiscoursal perspective (Hyland, 2005) or within the appraisal framework (Martin and White, 2005). The model proposed by Hyland (2005) distinguished between “interactive” (textual) and “interactional” (interpersonal) functions of metadiscourse in a text. Interactive metadiscourse primarily involved the “management of information flow,” whereas interactional metadiscourse was “more personal” (Hyland, 2005, p. 44). In other words, the former concerned with orienting readers with the help of signposts such as frame markers, transition markers, sequencers, and code glosses, while the latter referred to the interaction-oriented expressions of attitudes, hedges, and boosters to engage readers more overtly by evaluating and commenting on the text. Interest markers, in essence, constitute a part of attitude markers and connect with the other types of interactional metadiscourse (i.e., hedges, boosters, and self-mentions) discussed by Hyland (2005). Linguistic evaluative resources manifested in expressions such as *interestingly* and *intriguing* could communicate authorial stances and attitudes in discourse. In addition, the intensity of this emotive response could be mitigated by hedges such as *somewhat appealing* or enhanced by boosters such as *very interestingly*. Moreover, self-mentions explicitly identify experiencers of this emotion, as revealed by such examples as *the authors were interested in.*

Apart from being addressed from the metadiscoursal perspective, interest markers were also examined within the appraisal framework consisting of three evaluative systems: attitude, engagement, and graduation, with each system being comprised of its own subcategories (Martin and White, 2005). Linguistic expressions of interest fall into the attitude system. Specifically, they are affective responses, as illustrated by *We were intrigued by the recent meta-analysis by Pilling et al. (2002)*. Furthermore, these markers can also indicate a judgment about people or behavior. For instance, *He is a very interesting person* conveys a judgment of the person’s character. Moreover, interest markers are also indicators of appreciation, for example, signaling our “reactions” as illustrated by *The results reported are very interesting.* Finally, they are also associated with the graduation system since these emotions can either be sharpened/upscaled or softened/downscaled, suggesting varying intensity levels. For example, “force” resources can be employed to enhance or mitigate the degree of evaluation, as illustrated by expressions such as *particularly interesting*. Similarly, they can also be used to indicate the prototypicality or centrality of a phenomenon or an attitude, as illustrated by the examples of *We are genuinely interested in,* and *These statistical analyses were really interesting*. Among the studies examining evaluative expressions in academic discourse, Tutin (2010), for instance, investigated the use of evaluative adjectives in French academic writing and found that authors from disciplines such as Linguistics and Economics tended to deploy these linguistic resources to provide justification for the author’s research and claim the significance of the results obtained. Moreover, in a study that analyzed evaluative adjectives as attitude markers in scholarly writing, Koutsantoni (2004) revealed that these expressions, especially when used positively, contributed to enhancing the originality of the author’s study.

As made clear by the discussion above, attitude markers such as expressing interest served primarily textual and interpersonal metafunctions of language (Halliday and Matthiessen, 2013) in the metadiscoursal and appraisal frameworks. Examining such explicitly used linguistic devices as functional markers within these frameworks indeed captured the interactive feature of writer-reader communication. However, it should be noted that these functionally oriented analytical approaches could not account for the key semantic features and cognitive properties of a specific type of attitude markers, i.e., interest markers, in academic writing. As noted earlier, interest markers are essential cognitive resources for fostering the creation and growth of knowledge (Silvia, 2019). Therefore, to better understand how these markers relate to knowledge construction, a fine-grained and semantically oriented conceptual framework is needed to capture their semantic properties in academic writing. To this end, frame semantics provides the needed apparatus for accomplishing the task.

### Frame semantics as a cognitive semantic approach to academic writing

Frame semantics (Fillmore, 1985; Fillmore and Baker, 2010) proposed by Fillmore could provide a powerful conceptual tool for developing semantic frames for an understanding of the use of interest markers. This linguistic theory, deemed a cognitive linguistic framework of language understanding, assumes that a word’s meaning is construed in relation to our background knowledge acquired from different types of previous experience. Fillmore (1985) held that a semantic frame is a script-like coherent structure of concepts, which constitutes a schematic representation of an event, a particular situation, or a relation. Fillmore provided the Commercial_transaction frame depicting participation in a scenario of commercial transactions with different roles to exemplify what a frame is. This frame includes roles such as *buyer* (someone who has money and wants to exchange it with goods), *seller* (someone who has goods and wants to exchange it for money), *goods* (the item that is exchanged for money), and *money* (any circulating medium of exchange, including coins or paper money). The buyer yields money and takes the goods, and the seller yields the goods and takes the money. Frame elements (FEs) are the roles (i.e., buyer, seller, goods, and money), participants, props, and conceptual elements that constitute the frame. Informed by frame semantics, the Berkeley FrameNet research project (Baker et al., 2003; Ruppenhofer et al., 2016) is a unique online database documenting a wide variety of frame semantics descriptions and syntactic information for the core English lexicon.

According to Ruppenhofer et al. (2016), FEs can be classified into core and peripheral elements “in terms of how central they are to a particular frame” (p. 23). “A core frame element instantiates a conceptually necessary component of a frame while making the frame unique and different from other frames” (Ruppenhofer et al., 2016, p. 19). In other words, core frame elements could uniquely define a frame and capture the essential aspects of an evoked frame. For example, in the Commercial_transaction frame, *buyer*, *seller*, *goods*, and *money* are all core elements of the frame because they are crucial to understanding the frame. The Commercial_transaction frame cannot exist without a buyer or seller. In contrast, peripheral frame elements relate to those that characterize the scene more generally, such as the medium, time, degree, or place when an event occurs (Ruppenhofer et al., 2016). For instance, frame elements such as time, place, and degree in the Commercial_transaction frame are peripheral because they do not uniquely distinguish the frame but merely provide additional information. The given Examples 1–2, taken from the present corpus and presented in line with FrameNet annotation format, illustrate the Stimulus_focus frame with its FEs evoked by a typical interest marker, *intriguing* and the Experiencer_focused_emotion frame evoked by *interest* in applied linguistics RAs, respectively.

1. He also found a [_Degree_ Very] *INTRIGUING*^Target^ [_Stimulus_ sex difference in LLS use, with females showing a greater propensity than males to engage in out-of-class social interactions].
2. [_Experiencer_ researchers in language and education policy] have long been *INTERESTED*^Target^ in [_Content_ the theories and literature of LI to explain and even to predict the effectiveness of language policy in society] [_Explanation_ given that language planning has not only developed in depth, but also breadth which reached a hitherto unknown degree].

In line with the annotations given by FrameNet, Stimulus (*sex difference in LLS use…*) and Content (*the theories and literature of LI to explain and even to predict…*) are core frame elements suggesting what evokes this emotive response, whereas Degree (instantiated by *very*), Experiencer (*researchers in language and education policy*), and Explanation (*given that*…) are peripheral frame elements. A close examination of frame elements evoked by interest markers led us to conclude that FrameNet assigns different names to the conceptually equivalent FEs related to what triggers the feeling of interest. For example, in the examples presented above, Stimulus in the Stimulus_focus frame is renamed Content in the Experiencer_focused_emotion frame although they all indicate the causes of interest. Likewise, Explanation that indicates why something is interesting is labeled Circumstance in the Stimulus_focus frame but is renamed Reason in the Emotion_directed frame.

In addition to describing semantic frames and their FEs, FrameNet documents relations between semantic frames evoked by lexical units such as Inheritance, Perspective_on, and Using. Frame-frame relations allow “frames (and thus their lexical units) to be associated despite being separated” (Ruppenhofer et al., 2016, p.79), thereby connecting frames to constitute a network of their concepts. According to FrameNet, Inheritance relation describes a connection between a child and a parent frame in which a child frame has all the semantic characteristics and properties of a parent frame (Ruppenhofer et al., 2016). The Perspective_on relation indicates “the presence of at least two perspectives or different points of view on the Neutral frame (non-lexical and non-perspectivized)” (Ruppenhofer et al., 2016, p. 82). The Using relation involves “a particular frame making reference in a very general kind of way to the structure of a more abstract, schematic frame” (Ruppenhofer et al., 2016, p. 83). Apparently, such frame-frame connections and the conceptual overlapping of frame elements across related frames would make it possible to generate a generic semantic frame (Hu and Chen, 2019) for interest markers as an analytical framework to uncover how applied linguists’ geo-academic location might affect their deployment of these markers for communicating science. The Interest frame developed for the interest markers will be presented later.

### Geo-epistemological orientations and academic writing

The concept of geo-epistemology assumes that knowledge production and circulation cannot be detached from the immediate physical space involved (Agnew and Livingstone, 2011; Mignolo, 2012). Geo-epistemology well captures how geographical locations tend to influence the process of knowledge production and interpretation because of historical, academic, and cultural trajectories (Agnew, 2007). Our conceptual perspectives developed from the immediate environment cannot be detached from the local domain where we work or live. Of course, given the mobility of academics worldwide, the location where an author works may not necessarily match his/her nationality. For example, some academics affiliated with institutions situated in North America are nationals of Asian countries and vice versa. However, academic values, attitudes, and conventions are socially constituted and thus are possibly shaped by the local community in which academics conduct their research activities. In addition, universities and research institutes, as prestigious knowledge production sites, are supposed to contribute to endogenous societal development (Mansell, 2014). As such, authors’ institutional affiliations are suggestive of the local academic community to which they belong.

As revealed by some research, epistemological assumptions and paradigms developed in a local or national context where intellectual styles may vary could exert a considerable influence on the choice of language and rhetorical practices in scientific discourse (Bennett, 2014; Bondi, 2014). Indeed, substantial textual differences in research papers by American authors and their British counterparts were noted by Swales (1996). He likened British writers’ communication style to the “quick-quick-quick-repeat” style (starting with interesting ideas, followed by some vague methodology, scrappy results, and a summary), in contrast to their American counterparts’ preference for the “slow-slow-slow-quick” style (beginning with an exhaustive review of the extant literature, followed by painstaking methods, the results, and a thorough discussion; Swales, 1996, p. 46). Along the same line, Tight (2007) also found that higher education scholars across regions tended to have different referencing practices. Specifically, higher education scholars based in North America and Britain were prone to write without referring to any publications/policies coming from outside their systems whereas the scholars from other countries, for example, Australia and the Netherlands, showed an opposite preference (reference to studies/experience/evidence outside their system). Tight speculated that scholars working within a more extensive and distinctive higher education system would be more inward-looking, while researchers working within relatively smaller systems would be more inclined to situate their research in a more comparative or global context. In addition, Tight reported that North American researchers’ texts tended to describe theoretical and methodological issues more explicitly than scholars based in other countries and regions. In a study conducted by Yakhontova (2002), it was found that Ukrainian and Russian academic authors were inclined to employ more positive evaluative language in their abstracts than Western scholars.

Since having access to knowledge is a prerequisite for new knowledge creation (Graham et al., 2011), it is not surprising that researchers from the Center, namely the Anglophone and other European countries, have enjoyed a disproportionately large percentage of academic publications due to this kind of “academic imperialism” (Fewer, 1997, p. 764). Canagarajah’s (2002) study on the geopolitics of academic writing critically evaluated the Western textual conventions, publishing communities, and social norms governing academic writing, through which the forms of intellectual hegemony stemming from the linguistic dominance of English were unveiled. This study, therefore, called for a reconfiguration of the Western-dominated knowledge production market from Western-centered literacy to more democratic realms of scholarship. As Canagarajah (2002) pointed out, the production of scientific knowledge is ideological, value-ridden, and contextual. When science involves a Center-Periphery relation, it is almost impossible to separate knowledge from the location where it is produced. Notably, some research (Acharya, 2014; Wemheuer-Vogelaar and Peters, 2016; Alejandro, 2018) probed into the Western-dominated scholarly work in the discipline of International Relations (IR). For example, Wemheuer-Vogelaar and Peters (2016) revealed how the regional context shaped the academic practices of IR scholars, although they more often than not identified themselves with issue-based research communities crossing geographic boundaries. It was reported that Western scholars (e.g., United States, Canada, and Western Europe) were more likely to eschew traditional paradigmatic analysis in their publications, whereas non-Western researchers (e.g., Latin America or East Asia) were almost twice as likely to choose Marxism as a theoretical framework. Moreover, Western scholars’ strong preference for qualitative or quantitative methods in conducting research was observed, in contrast to the non-Western scholars’ propensity to conduct policy analysis. In addition, scholars from the West most likely identified themselves with the global community, while their non-Western counterparts predominantly opted for national or subnational communities in research practices.

In summary, previous work on the knowledge-making practices upheld by scholars across geographically defined regions has added to our understanding of scientific communication. However, research on the potential mediating effects of authors’ geo-academic location on their choice of interest markers in RAs for expressing evaluative attitude is rather limited. This study, therefore, set out to bridge the research gap and provide new insights into how the use of linguistic expressions of interest in scientific communication may be leveraged by an academic author’s geo-academic location.

## Materials and methods

### Corpus

To address the research questions, a corpus of 160 full-length applied linguistics RAs (1 million words) was compiled to examine how linguistically expressed interest was mediated by various contextual variables such as authors’ disciplinary background, gender, geo-academic location, and time of publication. These RAs were written by male and female scholars from core and peripheral regions of scientific research, and were published in two periods separated by a 30-year interval (1985–1989 vs. 2015–2019). The two publishing periods were chosen to examine possible diachronic changes in the use of these markers. The gender of the sole author or the first author of a co-author article was determined by capitalizing on multiple sources of information available (e.g., the bio-notes attached to the RAs, faculty profiles, Academia.edu, Researchgate, academic blogs, Facebook pages, and LinkedIn). The geo-academic location of the researcher in terms of his/her institutional affiliation was roughly divided into two broad regions: Anglophone countries + other Western and Northern European countries (Core) vs. the remaining countries (Periphery). This division was informed by Canagarajah (2002) and Kieńć (2017). The former study that examined inequalities in academic publishing referred to the West as center academic communities and those colonized by European invasion, i.e., the Third World, as periphery ones. The latter one defined scholars’ core and periphery status according to whether they were “based in countries with a gross domestic product (GDP) *per capita* less or greater than US$18,000″ (p.125). Kieńć (2017) used this classification because there was a strong co-relation (0.84) of a country’s publication output and GDP *per capita* according to World Bank Data on academic journal articles (Kieńć, 2017). Informed by these studies, Core regions in the present study refer to Anglophone countries as well as other Western and North European countries, such as the United States, Canada, Britain, Australia, Belgium, Czech Republic, Finland, Germany, and France. Periphery regions consist of those areas that do not fall into the Core category, such as Asian countries, Latin America, and Caribbean countries. The geographical location of the singe or the first author’s research affiliation was checked and categorized into Core-based academics or Periphery-based academics. Given the limited space, this paper only focused on how the use of interest markers might be mediated by an academic author’s geo-academic location. As presented in Table 1, these empirical RAs were randomly chosen from four prestigious journals that are identified with a high impact and nominated by disciplinary experts.

**Table 1:** Profile of the corpus.

Time	Geo-academic location	Gender	No. of RAs	Total no. of words	No. of words/RA	Journals included
Time 1	Core	Male	20	175,840	8,792	*Applied Linguistics*
		Female	20	173,860	8,693	*Language Learning*
	Periphery	Male	20	171,840	8,592	*TESOL Quarterly*
		Female	20	174,740	8,737	*The Modern Language Journal*
Time 2	Core	Male	20	98,640	4,932	*Applied Linguistics*
		Female	20	102,120	5,106	*Language Learning*
	Periphery	Male	20	97,880	4,894	*Foreign Language Annals*
		Female	20	99,320	4,966	*The Modern Language Journal*

### Text-based interviews

Text-based interviews in a semi-structure format were conducted with disciplinary specialists to complement the corpus-based quantitative analyses of interest markers found in the applied linguistics RAs. The interviews were intended to explore what motivated applied linguists to employ interest markers and their perceptions of other scholars’ use of these markers. The interview guide (Appendix A) purported to elicit the interviewees’ responses concerning their considerations for their use or non-use of these markers based on an extract authored by himself/herself. It allowed deviations, digressions, and expansions from the prompts so that both interviewer and interviewees were at liberty to raise questions of relevance coming up in the course of interviews (Mackey and Gass, 2016). The method that allows for the exploration of situated meanings of a text (Lillis, 2008) could provide insights into academic writers’ intentions behind their choice of interest markers. Hyland (2012) also remarked that gaining insights from “activities surrounding the production and reception of texts and how participants actually understand what they are doing with them” (p. 37) contributed to a greater understanding of academic writing as a socially negotiated act.

In total, 6 disciplinary informants from applied linguistics, identified based on the corpus constructed for this study, were enlisted (see Table 2). Each interview lasted approximately 30 mins and was conducted in English or Chinese (the first language of some interviewees). The transcripts were sent to the informants to check for accuracy after the interviews were transcribed. The interviewees were referred to as Informants 1, 2, and so on to preserve anonymity. All of them had a considerable number of English publications in prestigious journals. The interview results were used to triangulate the quantitative findings obtained from the corpus-based analysis.

**Table 2:** Demographic information of participants for the interview.

Informant	Location	Academic rank
I-1	Core (United States)	Professor
I-2	Periphery (China)	Associate professor
I-3	Core (Britain)	Associate professor
I-4	Periphery (Portugal)	Assistant professor
I-5	Periphery (Russia)	Professor
I-6	Core (Australia)	Assistant professor

### Analytical framework: The interest frame with its FEs

**Figure 1:**
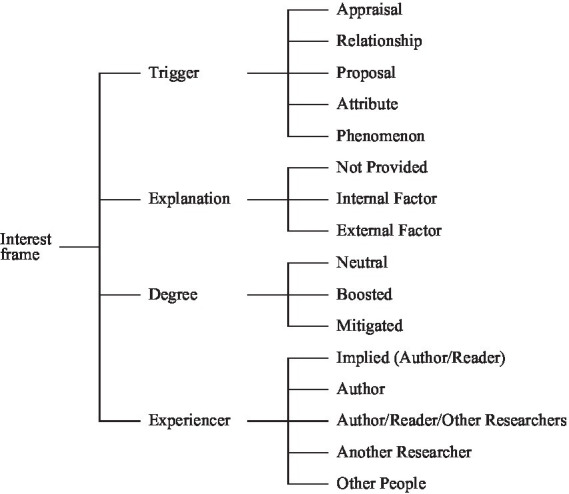
The interest frame (Wang and Hu, 2022).

The Interest frame developed by Wang and Hu (2022) was used as an analytical framework to code frame instances associated with interest markers in the corpus. The main procedures for developing this frame are briefly presented. A lexical approach (Hu and Chen, 2019) was adopted to generate a generic Interest frame for the RAs sampled. Informed by Thesaurus.com and *Thesaurus by Merriam-Webster,* a list of headwords that were either synonyms or antonyms of interest and all its derivative forms were compiled. This list of words was used as search words (Appendix B) to identify interest markers in our dataset. All the hits in the corpus were manually checked to remove those lexical items irrelevant to the expressions of interest, such as *interest* in *raising interest rates.* These markers were scrutinized and found to evoke six interconnected semantic frames *via* frame relations of Using, Inheritance and Perspective_on described by FrameNet. The six semantic frames are the Emotion-directed frame, the Stimulate_emotion frame, the Experience_focused_emotion frame, the Stimulate_focus frame, the Stimulate_emotion frame, and the Mental_stimulus_exp_focus frame, respectively. Then, a coding scheme based on the FEs associated with these semantic frames that FrameNet lists was developed. The coding scheme was used to code and identify all the FEs of the interest markers that occurred in the corpus. It was found that Stimulus occurred most frequently, followed by Degree, Experiencer, and Explanation. As noted earlier, the interconnections of the interest-related frames and the conceptual overlapping among frame elements across various frames made it possible to generate the Interest frame (Figure 1). In what follows, the illustration of each frame element and its subcategories are presented in detail.

Trigger, as a core frame element, indicates what elicits the interest. The first type of Trigger, Appraisal, refers to evaluations of the value, significance, or implications of the findings or results of the current study or previous studies (Example 3). The second type of Trigger includes relationships between different research variables or objects, between results obtained in the current study or from different studies, as shown in Example 4. The third type of Trigger, Proposal, includes hypotheses, viewpoints, or potential research trends suggested or proposed (Example 5). Attribute, as another type of Trigger, concerns the distinctive features, characteristics, and qualities of research methods, research objects, research variables, or participants, as seen in Example 6. Finally, as a Trigger for the emotion of interest, Phenomenon refers to experiences, entities, and happenings that do not fall into the categories mentioned above, as illustrated by Example 7. In the corpus of applied linguistics RAs, the five categories of Trigger (in the order presented above) accounted for 25%, 14%, 16%, 13%, and 32% of the frame instances, respectively.

3. This finding suggests a very *INTRIGUING*^Target^ [_Trigger_ possibility that increased cognitive task complexity might be associated with diminished L1 influences on comprehensibility].
4. The relationship between L2 listening and the ability to discriminate consonants was the highest for these learners (about *r* = 0.37). As the authors suggest, these results indicate an *INTERESTING*^Target^ [_Trigger_ relationship]; however, this is not necessarily a causal relationship.
5. [_Trigger_ Examining learners’ motivations for maintaining the gains made abroad as well as their self-perceptions of language maintenance] would be *INTERESTING*^Target^ areas to explore in future research to better understand to what extent we are meeting the needs of these students in order to maintain the gains they made while abroad.
6. This study has clear limitations: due to space constraints, many *INTERESTING*^Target^ [_Trigger_ data episodes] are omitted.
7. Perhaps *INTERESTINGLY*^Target^, [_Trigger_ no mention is made of any difficulty in learning two typologically different languages concurrently].

Explanation concerns the reason for the evoked feeling of interest. Some research has shown that people find something interesting when they appraise events as more relevant to them (Connelly, 2011) or perceive an event as novel, unexpected, complex, yet potentially comprehensible (Reeve et al., 2015; Silvia, 2019). The factors contributing to feeling interested could be signaled explicitly or implicitly in scientific communication. When an explanation is provided explicitly, the elicited interest could be ascribed to internal or external factors. The internal ones relate to results or findings from the current study or characteristics of research objects, variables or participants, as shown in Example 8. The external factors refer to hypotheses, the results, and findings of previous research or characteristics of the research background or context, as illustrated by Example 9. In the corpus, most frame instances (77%) did not explain why something was interesting (see Example 10), while 15% provided explanations related to external factors, and 8% gave explanations related to internal factors.

8. The latter finding is particularly *INTERESTING*^Target^, [_Explanation_ as the vocabulary test was very easy (see Appendix C): Learners who failed to achieve the maximum score on L1 vocabulary were likely to be among the weakest FL readers].
9. This finding obtained from Wicherts et al. (2011) is *INTERESTING*^Target^, [_Explanation_ given the previous evidence that researchers who were more willing to share their data were less likely to have committed errors in reporting their results].
10. However, these studies also reveal some *INTERESTING*^Target^ inconsistencies indicative of a nascent drive toward vindication of English spoken with Chinese accents.

Degree concerns the intensity of an expressed interest and describes how strongly it is felt. The emotion could be boosted (Example 11) or mitigated (see Example 12). Most of the frame instances (i.e., 71%), however, fell into the “neutral” category, with the expressed interest being neither boosted nor weakened (Examples 13), compared with the 21% that were boosted and the 8% that were mitigated. Notably, when expressed interest was boosted, various boosting devices were employed, for example, *very*, *certainly,* and *particularly*. When expressed interest was hedged, *perhaps*, *may* and *somewhat* were preferred.

11. This finding suggests an [_Degree_ very] *INTRIGUING*^Target^ possibility that increased cognitive task complexity might be associated with diminished L1 influences on comprehensibility.
12. [_Degree_ Perhaps], a *FASCINATING*^Target^ but little-understood aspect of language learning is the involuntary processing of language features.
13. It is *INTRIGUING*^Target^ that learner motivation in the experimental group did not improve in motivational intensity as one important aspect of motivation.

Experiencer refers to the person who experiences the emotion of interest. Experiencers can either be stated or implied in RAs. It was found that the omission of the Experiencers was salient, occurring with a majority of cases of expressed interest (69%), as shown in Example 14. When the experiencers were explicitly provided, five different categories were identified, including Author(s) (15% of instances of expressed interest), as seen in Example 15, Author/Reader/Other Researchers (7%), as shown in Example 16, Another Researcher (4%), as illustrated by Example 17, and Other People (5%), as illustrated by Example 18.

14. The assumption of a phonological core deficit in FL, though *APPEALING*^Target^, has to be evaluated with further investigation.
15. [_Experiencer_ We] were particularly *INTERESTED*^Target^ in LLS teachability.
16. It is obvious that they must draw on the insights and methodologies of [_Experiencer_ linguists, sociologists, and practitioners of other disciplines] who have long been *INTERESTED*^Target^ in the phenomenon of cultural diffusion and transformation.
17. [_Experiencer_ Wicherts et al. (2006)] were *INTERESTED*^Target^ in reanalyzing data from published research in psychology to examine the sensitivity of reported findings to outliers.
18. However, as [_Experiencer_ Jane] was only interested in two ranks, the fact that 50 instead of 57 judges participated in the test did not detract from the power of the test or the validity of the conclusion.

### Methods of data coding and analysis

Drawing on the Interest frame presented earlier, two coders (the author and trained coder with a doctorate in applied linguistics) coded all the frame instances of interest markers in applied linguistics RAs independently. All disagreements were resolved through discussion. The results for inter-rater reliability showed Cohen’s Kappa values of 0.79 with a 95% confidence interval (CI) from 0.68 to 0.85 for the frame element of Trigger, of 0.76 with a 95% confidence interval (CI) from 0.74 to 0.88 for Explanation, of 0.85 with a 95% confidence interval (CI) from 0.77 to 0.82 for Degree, and of 0.85 with a 95% confidence interval (CI) from 0.77 to 0.82 for Experiencer, respectively. These values indicated good inter-coder reliability (Hallgren, 2012).

To address the RQs of this study, binary logistical regression analyses were conducted using SPSS 23.0. Such analyses were intended to determine whether the predictor variable, that is, the author’s geo-academic location could predict the absence or presence of the Interest frame elements and their subcategories in an applied linguistics RAs. Given the binominal measures in this study, the outcome variables were coded as dichotomous variables, i.e., the absence or presence of an interest marker and its frame elements. The reasons for using logistical regression analyses were twofold: (1) there were not many instances of interest markers across the data; (2) the interest markers that occurred multiple times in the corpus shared the same source of incongruence.

While a binary logistical regression can provide the statistical results related to the choice of interest markers and their frame elements, Nagelkerke’s adjusted *R*^2^ and odds ratio indicators can index the proportion of variance explained by the predictor variable. The odds ratio indicating the likelihood of the occurrence of one event compared to another assumes a positive relationship between the two events if it is greater than 1. By contrast, a negative relationship can be assumed if an odds ratio is smaller than 1. In the binary logistical regressions, Core-based academics were set as the reference value. Bonferroni correction was applied to adjust the alpha value because multiple statistical tests were conducted on the subframes of interest markers.

For the interview data, a thematic analysis was conducted since this method illustrates the data in detail and addresses diverse subjects *via* interpretations (Boyatzis, 1998). As such, an in-depth analysis with the main focus either on the perspectives of separate or groups of individuals can be achieved by investigating the observational data emanating from participants’ opinions or feedback (Creswell and Creswell, 2017). All the transcribed data were uploaded to MAXQDA Pro (version 2018) and read through to generate prominent themes related to the research questions of this study. I started by generating initial codes and then grouped them by assigning them headings. Next, I compared, reexamined and revised the codes to identify the themes that emerged. The themes were then re-read, refined and finalized. The experts relevant to the RQs were included in this paper.

## Findings

### Overall distribution of interest markers by authors’ location

As shown in Table 3, the binary logistic regression on the overall distribution of interest markers in the corpus returned a statistically significant difference (*B* = −1.231, *p* < 0.001, Nagelkerke *R^2^* = 0.112, OR = 0.622). The full model explained approximately 11% variance in the outcome variable. The odds ratio statistics indicated that applied linguists from the Periphery regions were 1.6 times (dividing odds ratio by 1) more likely to employ interest markers in their RAs than those from the Core regions.

**Table 3:** Results of binary logistic regression on the overall use of interest markers.

Outcome	Predictor					Odds ratio (OR)	95% CI for OR	
		*B*	*SE*	*Wald*	*p*		Lower	Upper
Interest markers	Core vs. periphery	−1.231	0.336	2.631	0.000	0.622	1.721	8.045
	Constant	1.463	0.624	3.928	0.036	1.073		
	*R*^2^ = 0.069 (Cox and Snell); *R^2^* = 0.112 (Nagelkerke)							
	*Model χ^2^*(1) = 8.594, p < 0.001							

In the interviews, when the informants were asked about their opinions regarding the use of English for international publishing, the respondents agreed that scholars based in the Periphery had linguistic disadvantages compared to those from the Core. However, they also added that this linguistic handicap was not a decisive influence on the rejection of a paper. A Core-based scholar (I-6) commented that academic writing was also challenging to native speakers, so they did not believe that this linguistic superiority would make it easier for them to publish. Although a majority of the Periphery-based scholars expressed their linguistic concerns about using English to write up manuscripts by commenting that “English norms somehow are different (I-2),” they acknowledged that English publications allowed them to be more visible in the globalized research community. Informants 2 and 4, two Periphery-based scholars elaborated as follows:

Using English is something we cannot change… I feel it is sometimes difficult to achieve persuasiveness when writing in English. You basically have to restructure everything when you write in another language…; but English publications allow me to reach a wider audience and get more recognition…

In Portuguese, I can always find the words I like. I know if these words are accurate or not. However, when writing in English, I sometimes do not say what I know, only what I can.

Apart from the more general language limits, the academics in the Periphery had worries regarding the appropriate use of interest markers in writing. For example, informant 5 commented on his use of interest markers (Example 7 presented earlier) and stated that “it was not easy for him to decide if hedging should be expressed” because he was not sure “if the editors thought this was interesting, too.” Similarly, although informant 4 believed that these expressions were helpful to “draw the readers’ attention to that important information,” and thus increased “the possibility to get the paper published,” she was sometimes hesitant to employ the linguistically expressed interest since “frequent use of them may sound too emotional or make the study less rigorous.” Her responses echoed with comments given by informant 2 on fewer possibilities of publishing in top-tier journals by Periphery-based scholars.

When prompted for probable variations in the use or non-use of these markers by scholars across the Core and Periphery areas, the respondents stated that by intuition they were unable to discern any distinctions by stating that “we all expressed interest when we found something different or new.” However, when asked why they employed these markers, Periphery-based scholars emphasized their intention to “sell their work” (I-4) or “promote the significance of the study” (I-2). As informant 5 elaborated:

This linguistic choice was a kind of promotional tool to increase the likelihood of making my research visible. Comparatively speaking, it is not easy for us to get published in top journals. If you want to persuade editors that the study is good enough, you need to show that.

Notably, Periphery-based informants expressed their preference for collaborations with Core-based scholars to improve the quality of presentation because co-authorship provided great opportunities for them to learn how to make their manuscripts grammatically correct, stylistically acceptable, and rhetorically persuasive.

### Distributions of interest frame elements by authors’ geo-academic location

#### Trigger

As summarized in Table 4, the binary logistic regressions run on the FE of Trigger only located statistically significant differences in the subcategories of Proposal (*B* = 0.823, *p* < 0.001, Nagelkerke *R^2^* = 0.257, OR = 2.791) with the full model explaining impressively 26% of the variance in the outcome variable. The odds ratios showed that authors from the Periphery regions were 2.8 times more likely to express interest triggered by newly proposed hypotheses or potential research trends.

**Table 4:** Results of binary logistic regressions on the frame element of trigger.

Outcome	Predictor					Odds ratio	95% CI for odds ratio	
		*B*	*SE*	*Wald*	*p*		Lower	Upper
Appraisal	Core vs. periphery	−1.625	0.437	1.775	0.022	0.794	1.266	7.323
	Constant	−0.217	0.323	4.155	0.144	1.171		
	*R*^2^ = 0.015 (Cox and Snell); *R*^2^ = 0.028 (Nagelkerke)							
	Model *χ*^2^(1) = 9.116, *p* = 0.033							
Relationship	Core vs. periphery	1.013	0.455	5.156	0.019	1.917	0.553	4.213
	Constant	2.116	0.645	9.116	0.073	1.653		
	*R*^2^ *= 0.*176 (Cox and Snell); *R*^2^ *= 0*.255 (Nagelkerke)							
	Model χ^2^(1) = 11.245, *p* = 0.081							
Proposal	Core vs. periphery	0.823	0.336	8.115	0.000	2.791	0.554	7.146
	Constant	−1.212	0.515	6.228	0.123	0.321		
	*R*^2^ = 0.121 (Cox and Snell); *R*^2^ = 0.257 (Nagelkerke)							
	Model *χ*^2^(1) = 10.807, *p* < 0.001							
Attribute	Core vs. periphery	1.773	0.427	6,355	0.051	2.103	0.666	6.185
	Constant	−1.316	0.553	9.535	0.226	0.721		
	*R*^2^ = 0.021 (Cox and Snell); *R*^2^ = 0.045 (Nagelkerke)							
	Model *χ*^2^(1) = 6.116, *p* = 0.082							
Phenomenon	Core vs. periphery	2.325	0.611	7.125	0.015	1.926	0.995	4.577
	Constant	−1.544	0.444	8.544	0.655	0.444		
	*R^2^* = 0.125(Cox and Snell); *R^2^* = 0.223 (Nagelkerke)							
	*Model χ^2^*(1) = 13.165, *p* = 0.071							

Informed by such results, Informant 3, a scholar from the Core regions, was asked to explain why her interest was triggered by a potential research issue (Example 5), she emphasized her intention of “putting forward a new question to get the readers’ attention” since she was “working on a new project related to that topic.” According to her, this was “another way to enhance the significance of her study.” Informant 1, a Core-based scholar also commented:

It is important to extend and build on your previous research, so you can become a kind of expert in the field. It is better to follow up what you have done….If you want to show you are a credible disciplinary member who knows this field quite well, you are obliged to give directions for this line of work…

#### Explanation

As presented in Table 5, the binary logistic regressions run on Explanation only found a significant association between geo-academic location and the incidence of unidentified sources of interest (*B* = 1.752, *p < 0*.001, Nagelkerke *R^2^* = 0.3123, OR = 1.911). The full model accounted for about 31% of the variance in the outcome variable. The odds ratio revealed that authors from the Core regions were 1.9 times more likely to leave the source of expressed interest unidentified than their counterparts from the Periphery regions.

**Table 5:** Results of binary logistic regressions on the frame element of explanation.

Outcome	Predictor					Odds ratio	95% CI for odds ratio	
		*B*	*SE*	*Wald*	*p*		Lower	Upper
Unidentified	Core vs. periphery	1.752	0.462	5.626	0.000	1.911	0.842	7.286
	Constant	−2.026	0.515	2.718	0.071	0.633		
	*R*^2^ = 0.221(Cox and Snell); *R*^2^ = 0.312 (Nagelkerke)							
	Model *χ*^2^(1) = 9.556, *p* = <0.001							
Internal factor	Core vs. periphery	2.073	0.453	5.262	0.052	1.882	1.255	6.313
	Constant	1.157	0.227	4.289	0.028	2.011		
	*R*^2^ *= 0.*016 (Cox and Snell); *R*^2^ *= 0*.026 (Nagelkerke)							
	Model χ^2^(1) = 9.463, *p* = 0.012							
External factor	Core vs. periphery	−1.746	0.423	6.185	0.022	0.825	1.937	8.226
	Constant	−2.628	0.682	9.268	0.055	0.367		
	*R^2^* = 0.161 (Cox and Snell); *R^2^* = 0.336 (Nagelkerke)							
	*Model χ^2^*(1) = 11.215, *p* = 0.052							

In light of the above quantitative results, informant 4, a Periphery-based researcher, was asked to comment on her intention to provide explanations for her expressed interest in her extract (Example 9). She opined that explaining why she evaluated the information as interesting would help target readers better understand the message and work toward a common understanding:

I believed that providing explanations for this emotion could make the message clearer because it helped invite my readers into the disciplinary dialog, so they would get along with my point…

Conversely, applied linguists from the Core regions, for example, informant 3, opined that “explanations were not necessary” because scientific research was supposed to “develop a specific field you know much about.” In the discipline of applied linguistics, “many people have been working in this field for quite a long time,” so that they have been “familiar with previous research.” In addition, informant 1 added that “the absence of an explanation would contribute to negotiating readers’ expectations” because “this gave them the impression that they were disciplinary experts and they could totally get the information.” Similarly, informant 6 also held that his readers could “see the same thing” as he did in the claims, so the omission of explanations would “help readers delve in the information encountered.”

#### Degree

For this frame element, Table 6 shows that geo-academic location significantly predicted the boosting of expressed interest in applied linguistics RAs (*B* = 1.963, *p < 0*.001, Nagelkerke *R^2^* = 0.322, OR = 3.062). The Nagelkerke *R*^2^ statistics indicated that the full model explained about 32% of the variance in the outcome model. Researchers from the Core regions were 3.1 times more likely than their counterparts from the Periphery regions to intensify their feeling of interest.

**Table 6:** Results of binary logistic regressions on the frame element of degree.

Outcome	Predictor					Odds ratio	95% CI for odds ratio	
		*B*	*SE*	*Wald*	*p*		Lower	Upper
Neutral	Core vs. periphery	2.114	0.426	2.551	0.472	2.011	1.035	8.257
	Constant	−2.735	0.546	5.216	0.268	0.785		
	*R*^2^ = 0.027 (Cox and Snell); *R*^2^ = 0.055 (Nagelkerke)							
	Model *χ*^2^(1) = 11.737, *p* = 0.039							
Mitigated	Core vs. periphery	1.316	0.428	5.219	0.214	1.827	0.822	6.212
	Constant	2.657	0.528	7.228	0.153	2.198		
	*R*^2^ *= 0.*008 (Cox and Snell); *R*^2^ *= 0*.125 (Nagelkerke)							
	Model χ^2^(1) = 10.678, *p* = 0.338							
Boosted	Core vs. periphery	1.963	0.586	5.218	0.000	3.062	1.736	8.328
	Constant	−2.776	0.474	7.136	0.055	0.463		
	*R^2^* = 0.186 (Cox and Snell)*; R^2^* = 0.322 (Nagelkerke)							
	*Model χ*^2^(1) = 12.643, *p* < 0.001							

When my Core-based informant (I-3) was asked to comment on the use of boosting expressions of interest instantiated by *very* (Example 11), she held that “presenting information with full assurance helped construct an authoritative disciplinary expert and this contributed to persuasiveness.” Similarly, informant 1 was convinced that the use of boosting conveyed the researchers’ confidence in their knowledge claims and researchers “had the need to argue for what they had found.” Different from the Core-based academics’ beliefs in the importance of making a stronger argument, the Periphery-based scholars thought that it would be better to “avoid being overconfident” (I-2) and “be cautious” (I-4) because that would probably “induce disagreement or doubts” (I-5) from the readers.

#### Experiencer

The statistical analyses (see Table 7) revealed a significant location-based difference for the subcategories of Implied (*B* = −1.116, *p < 0*.001, Nagelkerke *R^2^* = 0.205, OR = 0.558) and Author (*B* = 1.051, *p < 0*.001, Nagelkerke *R^2^* = 0.094, OR = 1.878). Scholars from the Periphery regions were 1.8 times more likely to leave Experiencers implied than their Core-based counterparts, while the latter group of researchers were 2.3 times more likely to describe themselves as people who experienced the expressed interest.

**Table 7:** Results of binary logistic regressions on the frame element of experiencer.

Outcome	Predictor					Odds ratio	95% CI for odds ratio	
		*B*	*SE*	*Wald*	*p*		Lower	Upper
Implied	Core vs. periphery	−1.116	0.472	3.276	0.000	0.558	1.372	8.166
	Constant	−2.826	0.614	2.148	0.024	0.512		
	*R*^2^ = 0.113(Cox and Snell); *R*^2^ = 0.205 (Nagelkerke)							
	Model *χ*^2^(1) = 6.264, *p* < 0.001							
Author	Core vs. periphery	1.051	0.322	5.218	0.000	2.278	0.939	7.262
	Constant	2.842	0.515	7.219	0.008	1.764		
	*R*^2^ *= 0.*025 (Cox and Snell); *R*^2^ *= 0*.089 (Nagelkerke)							
	Model χ^2^(1) = 13.815, *p* < 0.001							
Author/reader/other researchers	Core vs. periphery	−1.819	0.428	6.917	0.022	1.912	1.325	8.105
	Constant	−2.738	0.528	8.021	0.133	0.725		
	*R*^2^ = 0.015 (Cox and Snell); *R*^2^ = 0.033 (Nagelkerke)							
	Model *χ*^2^(1) = 5.384, *p* = 0.221							
Another researcher	Core vs. periphery	−1.074	0.538	8.155	0.033	0.928	1.038	5.256
	Constant	−2.768	0.628	9.226	0.318	0.022		
	*R*^2^ = 0.022 (Cox and Snell); *R*^2^ = 0.035 (Nagelkerke)							
	Model *χ*^2^(1) = 11.755, *p* = 0.081							
Other people	Core vs. Periphery	1.027	0.668	7.482	0.092	2.767	0.921	8.355
	Constant	−2.718	0.568	8.106	0.115	0.236		
	*R*^2^ = 0.045 (Cox and Snell)*; R^2^* = 0.067 (Nagelkerke)							
	*Model χ^2^*(1) = 12.645, *p* = 0.052							

In the interviews, when informant 4, a scholar based in the Periphery regions, was asked why he opted for the omission of experiencers (Example 14), he held that researchers should “stand behind” their work because persuasiveness might be achieved by “presenting the data and letting your argument talk.” By contrast, his Core-based counterpart, informant 1 opined that it was essential to “let readers know this was the author’s unique perspective” although he agreed that scientific writing needs to be “objective and faceless.” Notably, he associated his intrusion of the text by using *we* (Example 15) with the purpose of “establishing a credible disciplinary persona or image” and “better involving readers to co-construct knowledge.”

## Discussion

### Researchers’ overall use of interest markers across different regions

Our quantitative findings showed that academic authors based in Periphery research locations were inclined to use more interest markers for expressing their evaluative attitudes in applied linguistics RAs. This observation, perhaps, could be attributable to the Periphery-based scholars’ unequal access to and marginalization in the knowledge production market. Collyer (2018) noted that there exist staggering inequalities in global academic knowledge production, especially when English has become the default language for academic dissemination and communication (Hyland, 2015; Lillis and Curry, 2018; Mauranen et al., 2020). Consequently, the non-Center participants of the international academic community are greatly disadvantaged in gaining more visibility, better recognition, and more professional credit through publishing in high-profile Center-affiliated journals. Gregson et al. (2003) also pointed out that the internationalization of knowledge was “inevitably caught up in a complex web of power relations that connect power and knowledge…; writings themselves are constitutive of, and not just reflective of, power-knowledge systems” (p. 6). In fact, linguistic imperialism or hegemony has been well documented in a body of literature (e.g., Hyland, 2001; Canagarajah, 2002; Pennycook, 2017), indicating that knowledge of science, in its modern meaning, is equivalent to colonial science. As a primary mode of communicating scientific knowledge, academic discourse is socially constructed and infused with power relations in the international context (Bennett, 2014). In such context, Periphery-based scholars have to invest considerably more effort to promote their research and persuade the “gatekeepers” of high-ranking journals to accept their research. Their propensity to employ interest markers to describe their research findings highlights the hierarchical structure of the field. These practices, according to them, seem to be strategies to increase the likelihood of making their research visible in the international sphere. In addition, the Periphery-based informants’ responses regarding their great willingness to collaborate with Core-based scholars to make the writing more persuasive and linguistically acceptable, as a matter of fact, accentuate the privileged and dominant position of the Core-based scholars in the knowledge production market. To increase the possibility to get their research published, it is understandable that the Periphery-based scholars have a greater need to employ more interest markers in their RAs to better promote their research.

### Regional influences on the occurrence of the interest frame elements

The corpus-based analysis signaled some distinctive regional knowledge-making practices. It was found that the Core-based applied linguists were more likely to express their interest toward Proposal, and preferred to leave sources of expressed interest unidentified. In addition, they were more likely to tone up their expressed interest compared with their Periphery-based counterparts. Finally, they were more inclined not to identify experiencers of the emotion than those from the Periphery regions. However, if they chose to provide experiencers, they opted to describe themselves as Experiencers of the emotion.

The reasons why scholars from the Core regions were more likely to express their interest elicited by the proposed new hypotheses or a potential research direction might be plausibly attributed to their easier access to and dominance of the knowledge production world. According to the comments of the informants, the Core-based scholars are at the pinnacle of the academic hierarchy (Moletsane, 2015). In contrast, researchers residing in the Periphery are likely to be denied access to material resources and experience a disproportionate representation (Salager-Meyer, 2008; Curry and Lillis, 2017). In the academic field where English-speaking voices predominate (Curry and Lillis, 2017), academics based in the Core regions dictate potential research trends and thus “play a key role in setting research agendas and determining what gets published” (Hyland, 2015, p. 72). Consequently, they tend to highlight their disciplinary expertise by proposing what type of issues warrant more scholarly attention. Academic writing, in this sense, may incorporate “diverse semiotic resources and ecological affordances” (Canagarajah, 2013, p. 6), and is intertwined with epistemological forms of power (Maton and Moore, 2010; Canagarajah, 2013).

Additionally, the Core-based academics’ propensity to leave the expressed interest unexplained indicate their scholarly practices of foregrounding the readers’ shared epistemological assumptions for knowledge negotiation. Conversely, authors from the Periphery held that explaining the expressed interest contributed to engaging readers in a more effective disciplinary dialog. In the interviews, authors from the Periphery regions opined that their linguistic constraints in using English as an additional language (Hanauer et al., 2019) motivated them to offer explanations to ensure language clarity and thus facilitate readers’ comprehension. In fact, this linguistic hegemony (Canagarajah, 2002; Pennycook, 2017) also suggests that academic discourse is infused with power relations in the globalized academia (Bennett, 2014).

The Core-based authors’ preference for intensifying their expressed interest probably relates to their epistemological beliefs that persuasion could be better achieved through constructing an authoritative persona by evaluating their arguments with a higher degree of assurance. As evidenced by the interview data, they emphasized the critical role of strong convictions could play in negotiating readers’ expectations for legitimating knowledge claims. Notably, they also highlighted the importance of constructing a more prominent authorial stance to claim their role as an arguer responsible for the propositional information. This knowledge-making practice could probably be ascribed to the Anglophone writing featuring a writer-responsible culture (Hinds, 2001) as well as the rhetorical function of authorial presence in establishing the author’s image as privileged disciplinary knowers (Hyland and Jiang, 2017), and promoting the research (Walková, 2019). Conversely, the Periphery-based scholars’ preference for authorial absence in scientific writing signals their understanding of scholarly ethos such as valuing objectivity. Consequently, depersonalization would be helpful to avoid imposing authors’ interpretations of arguments on readers (Yakhontova, 2006). As noted by Sabaj et al.’s (2013) study, compared to the “self-promotional” feature of scholarly writing in English (Hyland, 2001), Spanish scholars were convinced that the trait of modesty contributed to academic persuasion. This suggests that the locally valued discursive style of relying on research findings rather than authorial visibility held by Periphery-based academics may also impinge on academic texts. As noted by Mauranen et al. (2020), interdiscursive hybridity has become a prominent feature for academic writing in the globalization process.

## Conclusion

As a privileged academic avenue for knowledge production and dissemination, RAs are an essential channel to safeguard academic positions and prestige. This study, taking a cognitive semantic approach, examined how linguistic expressions of interest in applied linguistics RAs were leveraged by an academic author’s geo-academic location. It was found that scholars from the Periphery research communities were 1.6 times more likely to use interest markers in their RAs than their counterparts based in Core regions. Moreover, the two groups of scholars exhibited some location-based differences in knowledge-making practices through their deployment of some frame elements of interest markers. While the Periphery-based academics were 2.8 times more likely to express their interest triggered by newly proposed hypotheses or potential research trends, the Core-based academics were 1.9 times more likely not to identify the source of the expressed interest and were 3.1 times more likely than their Periphery-based counterparts to intensify their expressed interest. Finally, the latter group of researchers was 1.8 times more likely to leave the experiencers unidentified, whereas Core-based scholars were 2.3 times more likely to describe themselves as experiencers of expressed interest than those from the Periphery were.

The Periphery-based informants’ responses in the interviews revealed their concerns with linguistic drawbacks regarding using English to write up the manuscript. Given their disadvantaged position in the academia, they were incentivized to exert greater effort to claim the value of their study by employing more interest markers to boost their chances of publishing in top-ranked journals. Moreover, their preference for explaining the linguistically expressed interest index their disadvantaged position in gaining more visibility, better recognition, and more professional credit in the disciplinary community, compared to their Core-based counterparts. In contrast, Core-based academics who play the role of gatekeepers of high-profile journals, are privileged in scholarly publishing. Thus, they were prone to demonstrate a more confident, visible, and authoritative discoursal image in knowledge claims.

In summary, the examination of the mediating effect of authors’ geo-academic location on the use of interest markers has offered some insights into the possible connections of different geographically located research communities and their specific ways of constructing and communicating scientific ideas. However, this study has some limitations. First, it only focused on applied linguistics RAs, and it is worthwhile to examine cross-disciplinary variations in the use of interest markers and particularly, whether interdisciplinarity may impact the use of these markers as more trans-disciplinary and interdisciplinary research is emerging. Second, the adoption of a binary category, i.e., Core vs. Periphery, may obscure some intra-group distinctions since academic authors’ L1 background/nationality/ethnicity may also have some impact on their propensity to employ certain linguistic/rhetorical devices in knowledge claims. Finally, authors’ affiliations may not necessarily reflect locally defined knowledge-making practices due to academics’ increasing mobility. It would be potentially revealing to examine the interplay of various demographic factors such as researchers’ gender, cultural background, and the research communities to which they geographically belong. Such attempts are expected to yield a more in-depth understanding of the epistemic role that interest markers can play in knowledge construction and scientific communication.

## Data availability statement

The original contributions presented in the study are included in the article/Supplementary material, further inquiries can be directed to the corresponding author.

## Ethics statement

The project has been reviewed and approved by the PolyU Institutional Review Board (PolyU IRB; or its delegate board; Reference Number: HSEARS20211024001). The patients/participants provided their written informed consent to participate in this study.

## Author contributions

The author confirms being the sole contributor to this work and has approved it for publication.

## Funding

This work was supported by the Social Science Foundation of Shaanxi Province (Grant No. 2021K021).

## Conflict of interest

The author declares that the research was conducted in the absence of any commercial or financial relationships that could be construed as a potential conflict of interest.

## Publisher’s note

All claims expressed in this article are solely those of the authors and do not necessarily represent those of their affiliated organizations, or those of the publisher, the editors and the reviewers. Any product that may be evaluated in this article, or claim that may be made by its manufacturer, is not guaranteed or endorsed by the publisher.

## Supplementary material

The Supplementary material for this article can be found online at: https://www.frontiersin.org/articles/10.3389/fpsyg.2022.1020854/full#supplementary-material

## Footnotes

^1^The Core/Center was initially used to group counties that were geographically and economically advantaged in contrast to the Periphery which lacked the resources to engage in scholarly endeavors or knowledge-making practices (Canagarajah, 2002).
