# Supplementary material for: “The very interesting finding suggests that…”: A cognitive frame-based analysis of interest markers by authors’ geo-academic location in applied linguistics research articles
Source: Front Psychol. 2022 Oct 26;13:1020854. doi: 10.3389/fpsyg.2022.1020854 (PMC9644185; doi:10.3389/fpsyg.2022.1020854)
Supplement: Supplementary file 1 [file Data_Sheet_1.docx]

**Appendix A.** Interview Guide

1. What do you think about the use of English as the international language for scientific communication?
2. Do you think that the dominance of English in international publication gives an advantage to native speakers? If yes, in what ways？
3. Please look at this extract in which you have used interest markers. Why did you use this kind of expression?
4. Do you think your counterparts based in core/peripheral research institutions would use it in a different way?

**Appendix B** Search Words Used to Identify Interest Markers

| Category | Part of speech | Search word |
| --- | --- | --- |
| Interestingness | Verb | *interest, attract, absorb, captivate, entertain, fascinate,*  *grip, intrigue, involve, rivet, appeal to, arouse curiosity,*  *draw attention, grab attention, hold the attention of,*  *catch one’ s eye* |
|  |  |  |
|  |  |  |
|  | Noun | *interest, absorption, attentiveness, attention, attraction, appeal, captivation, curiosity, fascination, heed,*  *engrossment, inquisitiveness,* |
|  |  |  |
|  | Adjective | *interesting, interested, appealing, absorbing, arresting,*  *attractive, attracted, beguiling, captivating, curious,*  *compelling, engaging, engrossing, entertaining, enthralling,*  *enthusiastic, fascinated, gripping, intriguing, involving,*  *noteworthy, entrancing, enchanting, riveting, riveted, stimulated, stimulating, spellbinding, tantalizing,*  *obsessed, keen, eye-catching* |
|  |  |  |
|  |  |  |
|  |  |  |
|  | Adverb | *interestingly, appealingly, attractively, enthusiastically, keenly, fascinatingly, intriguingly, inquisitively, noticeably,*  *obsessively, tantalizingly* |
|  |  |  |
| Uninterestingness | Verb | *bore, disinterest, disregard* |
|  | Noun | *boredom, disinterestedness, unconcern, indifference* |
|  | Adjective | *boring, dull, dreary, monotonous, tiresome, tedious, weary, uninteresting, indifferent, unattractive, unconcerned, uninterested, unexciting, wearied* |
|  |  |  |
|  | Adverb | *uninterestingly, unattractively, indifferently, monotonously, tediously, wearily* |
|  |  |  |
